# Managing the global land resource

**DOI:** 10.1098/rspb.2017.2798

**Published:** 2018-03-07

**Authors:** Pete Smith

**Affiliations:** Institute of Biological and Environmental Sciences and ClimateXChange, University of Aberdeen, 23 St Machar Drive, Aberdeen AB24 3UU, UK

**Keywords:** climate change, land, food security, greenhouse gas removal, biodiversity, Sustainable Development Goal

## Abstract

With a growing population with changing demands, competition for the global land resource is increasing. We need to feed a projected population of 9–10 billion by 2050, rising to approximately 12 billion by 2100. At the same time, we need to reduce the climate impact of agriculture, forestry and other land use, and we almost certainly need to deliver land-based greenhouse gas removal for additional climate change mitigation. In addition, we need to deliver progress towards meeting the United Nations Sustainable Development Goals, all without compromising the many ecosystem services provided by land and without exceeding planetary boundaries. Managing the land to tackle these pressing issues is a major global challenge. In this perspective paper, I provide a very broad overview of the main challenges, and explore co-benefits, trade-offs and possible solutions.

## 1. The global challenges for which land management is critical

There are a number of global challenges that critically depend on the land if they are to be tackled successfully. These include food security [1], climate change mitigation [2–4] and the United Nations (UN) Sustainable Development Goals (SDGs) [5,6]. Specifically, the challenges are:

(1) *The UN SDGs*: In 2015, the UN defined 17 SDGs [5]: (1) no poverty, (2) zero hunger, (3) good health and well-being, (4) quality education, (5) gender equality, (6) clean water and sanitation, (7) affordable and clean energy, (8) decent work and economic growth, (9) industry, innovation and infrastructure, (10) reduced inequalities, (11) sustainable cities and communities, (12) responsible consumption and production, (13) climate action, (14) life below water, (15) life on land, (16) peace, justice and strong institutions, and (17) partnerships for the goals [5]. Of these, a number (particularly 2, 3, 6, 7, 8, 11, 12, 13, 14 and 15) have a significant reliance on land, so management of the land needs to be consistent with delivering these SDGs.
(2) *Food security*: The global population is projected to reach 9–10 billion by 2050 and approximately 12 billion by 2100. With more people moving out of poverty, there is a projected increase in demand for food in general, and livestock products in particular [7]. We need to provide more food on the planet in the next 50–80 years than has previously been produced in all of human history [8], on the same land base [9] and at the same time also reducing the environmental impact on farming [3,10,11].
(3) *Climate change mitigation*: The Paris Climate Agreement commits the 196 signatory countries to efforts to restrict climate warming to well below 2°C, with an aim to limit warming to 1.5°C above pre-industrial levels. With the agriculture, forestry and other land use (AFOLU) sector responsible for 24% of direct global anthropogenic greenhouse gas emissions, it is a major contributor to climate change [3]. On the other hand, there is potential in the sector to reduce greenhouse gas emissions and to provide sinks for greenhouse gases [2,3,12]. Across all sectors, these stringent targets are unlikely to be met without some form of atmospheric greenhouse gas removal (GGR) [4,13,14]. Many of the potential GGR options are land-based (e.g. soil carbon sequestration, biochar, bioenergy with carbon capture and storage, afforestation/reforestation and enhanced weathering of minerals) [4,15], and many have very significant land footprints and uncertainties [4,14,16].
(4) *Ecosystem services and planetary boundaries*: All of these challenges need to be met without compromising the ability of the land to deliver the many *ecosystem services* it provides, such as food, fibre and energy provision, provision of water, pollination services, climate regulation, nutrient cycling, hazard prevention, biodiversity and genetic resources, pollution control, the quality of soil, air and water, and delivery of cultural services (e.g. [17,18]), and without compromising *planetary boundaries*, such as those defined for climate change, ozone depletion, atmospheric aerosol loading, ocean acidification, nitrogen and phosphorus flows, freshwater use, land-system change and biosphere integrity (including functional and genetic diversity) [13,19].

Managing the land to tackle these pressing issues is a major global challenge. There are some co-benefits and some trade-offs associated with meeting these challenges. I discuss some of these below and examine how scientific knowledge can be used to deliver real-world solutions, before in the final section suggesting some options that have the potential to co-deliver on a range of fronts, with relatively few risks of adverse side effects.

## 2. Pathways to delivering global food security

We produce enough food on the planet to feed today's global population [1], yet over 800 million people go to bed hungry and undernourished each night [20]. Food insecurity needs to be dealt with not just by increasing production [21], but also by providing economic access to safe and nutritious food. It therefore requires action to improve distribution, governance, markets, access and infrastructure, among many other considerations [22]. Nevertheless, increasing food production sustainably will also be essential. One way to help deliver greater production is through the sustainable intensification of food production, by increasing productivity while reducing environmental footprint [10,23,24]. The aim is to increase the productivity of agriculture, while at the same time reducing the inputs and reducing the negative environmental externalities associated with production [25,26]. Sustainable intensification could also spare land [27], thereby freeing it for use for other purposes, such as land for conservation or land to produce bioenergy [2,27]. However, even sustainable intensification may not be enough to help deliver food security without adverse environmental impacts. Recent studies suggest that demand management is necessary, particularly through waste reduction [28–30] and dietary change [30,31]. In particular, current (and projected) levels of global overconsumption of livestock products cannot be sustained [31–33]. Recent studies [30] have shown that food security could be ensured and environmental impacts minimized if sustainable intensification (through yield gap closure) was accompanied by a shift to global healthy diets and a 50% reduction in food waste. Further studies have shown that demand management will be essential for transitioning to more sustainable agricultural production systems [34,35]. Demand management is therefore essential to ensure food security, but also has a valuable role to play in greenhouse gas emission reduction [2], as discussed in §3.

## 3. Pathways to delivering land-based climate change mitigation

Agriculture and forestry are responsible for approximately 24% of total human greenhouse gas emissions [2,3], and quantifying these emissions has been challenging [36,37], but the land sector also offers significant mitigation potential, through changes in land management that reduce greenhouse gas emissions or that create additional carbon sinks (e.g. soil carbon sequestration and afforestation) [2,3,12].

Greenhouse gas emissions from agriculture and forestry can be reduced through a range of management practices [3], including (i) reductions in CH_4_ or N_2_O emissions from croplands, grazing lands and livestock, (ii) conservation of existing carbon stocks (e.g. conservation of forest biomass, peatlands and soil carbon that would otherwise be lost), (iii) reductions of carbon losses from biota and soils (e.g. through management changes within the same land-use type, such as improved rotations, crops, tillage and residue management, or by reducing losses of carbon-rich ecosystems, such as reduced deforestation and rewetting of drained peatlands), (iv) enhancement of carbon sequestration in soils, biota and long-lived products through increases in the area of carbon-rich ecosystems such as forests (afforestation and reforestation), increased carbon storage per unit area (e.g. increased stocking density in forests), carbon sequestration in soils and wood use in construction activities, (v) changes in albedo resulting from land-use and land-cover changes that increase reflection of visible light, (vi) provision of products with low GHG emissions that can replace products with higher GHG emissions for delivering the same service (e.g. replacement of concrete and steel in buildings with wood and some bioenergy options), and (vii) reductions of direct emissions (e.g. agricultural machinery, pumps and fishing craft) or (viii) reductions of indirect emissions (e.g. production of fertilizers, emissions resulting from fossil energy use in agriculture, fisheries, aquaculture and forestry or from production of inputs), though indirect emission reductions are accounted for in the energy end-use sectors (buildings, industry, energy generation and transport) [3,12].

The economic mitigation potential (the potential that is cost-effective at given carbon price ranges) of all of these supply-side measures in the AFOLU sector combined is estimated to be 7.18–10.60 GtCO_2_-eq yr^−1^ in 2030 for mitigation efforts consistent with carbon prices up to 100 USD/tCO_2_-eq, about a third of which can be achieved at less than 20 USD/tCO_2_-eq [3]. Estimates from agricultural sector-only studies range from 0.3 to 4.6 GtCO_2-_eq yr^−1^ at prices up to 100 USD/tCO_2_-eq [2,3]. As mentioned in §2, demand-side options can also play a significant role in climate mitigation, in addition to their role in delivering food security. Among demand-side measures, which are under-researched compared to supply-side measures, changes in diet (largely a reduction in livestock product consumption) and reductions of losses in the food supply chain can have a significant, but uncertain, potential to reduce GHG emissions from food production (0.76–8.55 GtCO_2_-eq yr^−1^ by 2050), with the range being determined by assumptions about how the freed land is used [2,3].

**Figure 1.**
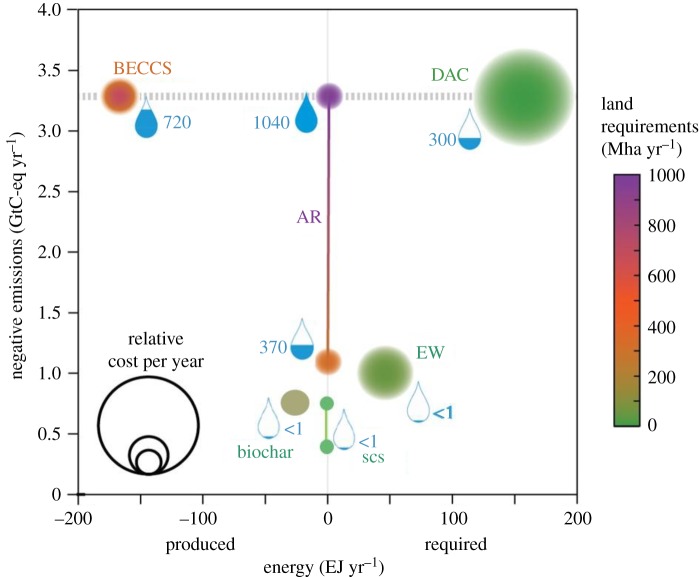
GGR potential for options direct air capture (DAC), enhanced weathering of minerals (EW), bioenergy with carbon capture and storage (BECCS), afforestation/reforestation (AR), soil carbon sequestration (SCS) and biochar and requirements for cost, energy, land and water [4,15]. GGR potential is shown on the *y*-axis and energy requirement (or energy generated) is shown on the *x*-axis. Land-use impact is shown by colour (see key). The size of the circle shows economic cost, and water requirement is shown in the water drop symbols, with quantities in km^3^ yr^−1^. All values are for 2100 except relative costs, which are for 2050 [4,15].

Recent analyses have suggested that even with aggressive and immediate mitigation action, it will be extremely challenging to meet the Paris targets through mitigation alone [4,14,38]. It appears that in addition to such mitigation, removal of greenhouse gases from the atmosphere will also be required. GGR could be achieved through engineering solutions (e.g. by direct air capture of CO_2_ and storage; DACCS) or through land-based solutions, for example by carbon storage in soils and vegetation, restoration of natural ecosystems, bioenergy with carbon capture and storage, addition of biochar to soils and the enhanced weathering of minerals [4,15,39]. Recent studies have shown that all available land-based GGR options have downsides, either through cost, energy, land, water or nutrient requirements or via physical climate impacts [4,15] (figure 1), though there are perhaps fewer downsides associated with some land-based measures [40]. Given the potential downsides, immediate and aggressive greenhouse gas mitigation action must remain the policy priority for tackling climate change, while R&D and demonstration projects could be used to remove barriers to deployment of GGR options [4,14].

## 4. Pathways to delivering the United Nations Sustainable Development Goals

**Figure 2.**
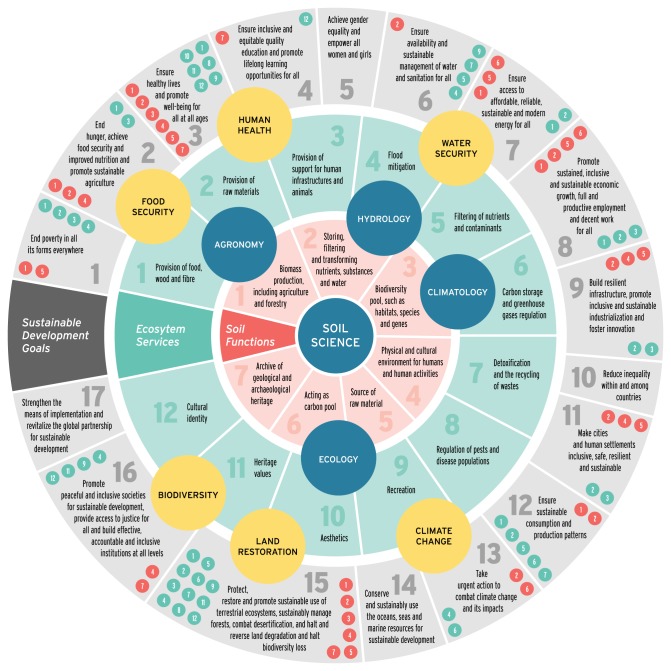
Links between soil science, soil functions, the ecosystem services they underpin and the 17 UN SDGs [6]. Soils are shown in the centre, with the functions they provide in the next circle. The next circle from the centre shows the ecosystem system services provided by these soil functions and the outermost circle shows the SDGs underpinned by the soil functions and ecosystem services. In the outer circle, for each SDG, the soil functions and ecosystem services that contribute to the delivery of each SDG are shown.

The management of land is involved with the delivery of most of the UN SDGs. For some, the link is clear (e.g. zero hunger requires food which requires land), while for others, the link is perhaps less obvious. Nevertheless, by mapping the functions provided by land/soils, and the ecosystem services they underpin—and then connecting these functions and ecosystem services with the delivery of each SDG, it is easier to conceptualize and quantify the role of land in delivering the SDGs [6]. For example, figure 2 presents a framework to map the role of soils onto the SDGs, by considering soil functions, the ecosystem services they underpin and how these functions and ecosystem services map onto each of the SDGs. Some related disciplines are shown as blue circles, and some of the global challenges are shown as yellow circles (figure 2). For each SDG, the soil functions and ecosystem service underpinning delivery of that SDG is show in the outer ring of the circle, with numbers keyed to each function/ecosystem service.

Among the SDGs, a number (particularly 2, 3, 6, 7, 8, 11, 12, 13, 14 and 15) have a significant reliance on land. As mentioned, zero hunger is directly reliant upon land from which the majority of human food is produced. Improved agricultural productivity will help to raise billions of people from poverty (SDG 1) and the sustainable nutrition provided will help to improve health and well-being (SDG 3). Land management (particularly restoration of wetlands) can help to provide clean water and sanitation (SDG 6) [40], and bioenergy from the land has the potential to provide affordable and clean energy [41,42]. Sustainable land management in and around cities can contribute to SDG 11 concerning sustainable cities and communities, and the demand-side measures discussed above for climate mitigation and food security will improve responsible consumption and production (SDG 12) [30]. The role of land in climate action (SDG 13) has already been discussed in §3 [2,3], while land management can affect life below water (e.g. by reducing erosion and runoff of pollutants) [43] and may impact positively or negatively on terrestrial biodiversity (SDG 15—life on land) [44–46]. Finding land management options that contribute to the delivery of the SDGs is therefore a priority when examining options to meet the global challenges discussed in this article.

## 5. Quantifying and managing synergies and trade-offs on land

**Figure 3.**
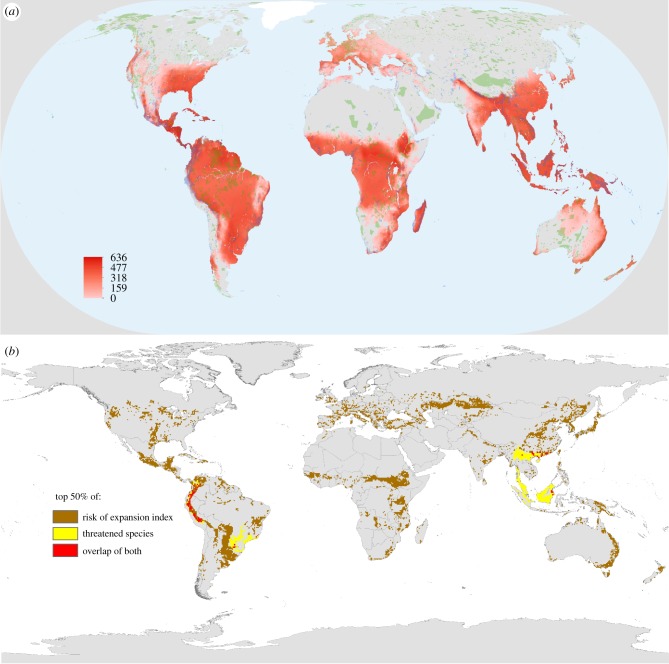
Potential trade-offs between biodiversity and (*a*) land-based renewable energy (in this case bioenergy) and (*b*) food security. In (*a*), overlap between power generation potential (GJ ha^−1^ yr^−1^; red colour gradient; see legend) for bioenergy (here represented by *Miscanthus x giganteus* as simulated by the MiscanFor model), constrained by energy demand, costs and carbon, overlaid with current protected areas (green shading) and global top 17% areas for protected area expansion (blue shading). Areas with no power generation potential are in grey. For bioenergy, no data were available for Greenland (adapted from [44]). Full details of the methods used can be found in [44]. In (*b*), the top 50% of threatened species richness and top 50% of risk of expansion index are plotted together, with areas shown in red where the areas overlap—showing a potential conflict between food security and biodiversity [48]. See [44,48] for full details.

Using ecosystem service modelling techniques developed in the mid-2000s [47], it has become possible to begin to assess synergies and trade-offs between different ecosystem services delivered by land. Recent assessments have included potential trade-offs between land-based renewable energy (wind, solar and bioenergy) and biodiversity [44,45] (figure 3*a*), and between the delivery of food security and biodiversity [48] (figure 3*b*). Using global estimates of the land requirements and energy generation potentials of the land-based renewable energy technologies such as wind, solar and bioenergy [49], the potential impact on biodiversity was assessed by examining the overlap between land most suitable for energy generation from each of the technologies, and current and projected protected areas [44,45]. Without restrictions on power generation, due to factors such as production and transport costs, bioenergy cultivation was found to be a major potential threat to biodiversity, while the potential impact of wind and solar appears smaller than that of bioenergy (bioenergy only shown in figure 3*a*). The differences are, however, reduced when energy potential is restricted by external factors including local energy demand. Overall, areas of opportunity for developing solar and wind with little harm to biodiversity exist in several regions of the world, with the magnitude of potential impact dependent on restrictions imposed by local energy demand [44]. Such analyses are useful for targeting global efforts for renewable energy development, climate mitigation and biodiversity conservation.

Trade-offs can also be seen between biodiversity and expansion of cropland for delivering food security [48]. There are a number of areas globally where the risk of agricultural expansion overlaps significantly with areas of threatened species richness (figure 3*b*). The study [48] showed that areas with both high biodiversity and high food insecurity or a high risk of agricultural expansion mainly occur in the tropics. The areas identified are especially at risk of biodiversity loss, highlighting the need to tackle the challenges of food insecurity and biodiversity loss together. A subsequent analysis [49] examined specific projections of cropland expansion from integrated assessment models, with a data on biodiversity hotspots, endangered and critically endangered species from various taxa, again highlighting potential future conflicts between use of land to address food security or to conserve biodiversity. Negative impacts on carbon storage (and thereby climate change mitigation) through cropland expansion were also identified [49].

The likely consequences of traditional land-based mitigation measures and land-based GGR options on biodiversity have recently been assessed [46]. The study concluded that efforts to meet a 1.5°C target through mitigation efforts would largely be consistent with biodiversity protection/enhancement depending on the mitigation approach used. However, additional effort to meet the 1.5°C target using some GGR technologies (e.g. soil carbon sequestration) would be neutral or positive, whereas others are likely to lead to biodiversity conflicts (e.g. bioenergy with carbon capture and storage) when applied at scales necessary for meaningful GGR. It was further noted that if GGR technologies are used to manage an overshoot of emissions/temperature increase, there could be additional direct impacts on biodiversity compared with those that do not overshoot, because temperature will be higher than 1.5°C for a period of time, so scenarios that avoid overshoot would have fewer adverse impacts than those that do not overshoot. Other land-based GGR options, such as afforestation/reforestation, are context-specific, but there is enough knowledge to implement these options in a manner that protects or enhances biodiversity, potentially offering adaptation benefits [49].

There is much more work to be done in assessing potential co-benefits and trade-offs among land management options to tackle different global challenges.

## 6. Helping to effect real-world change in practice

Important as identifying problems and proposing solutions are, effecting real-world change remains the greatest challenge. One way of effecting change is to make the best science available to decision-makers and land managers, for example through the developing software tools to improve real-world practice. One such software tool is the Cool Farm Tool (CFT) [50,51]. The CFT started life as a farm-based greenhouse gas calculator, which uses readily available farm management information (e.g. crops planted, fertilizer type, amount and timing, agrochemical application and timing, plant and harvest dates, livestock types, management and feed) to calculate greenhouse gas footprint per area, livestock unit or per unit of agricultural product [50]. It has been shown to perform very well against similar farm greenhouse gas calculators [52] and has since been further developed to calculate farm water footprint and biodiversity impact [53].

An example of the utility of the CFT is demonstrated in a case study of 10 large-scale egg producers in the USA (representing the entire supply of organic eggs to one large retailer), who used the CFT over 3 years to calculate their emissions [54]. The producers were trained to use the tool and calculated their greenhouse gas footprints. The highest emissions were found to be associated with feed, followed by transport and manure management. Through use of the tool, the farmers became aware of the sources of emissions in egg production. Though no targets for emission reduction were set, the farmers began to take action to reduce emissions, learning best practice from each other when comparing results. The results showed that GHG emissions were decreased over the 3 years of the study by approximately 15% (range 4–33% for individual farms) [54].

Since its initial development, the CFT has been adopted by an industry partnership including a number of the world's largest agri-food businesses (e.g. Danone, M&S, Kelloggs, Heineken, PepsiCo, McCain, Nestle, Unilever and Tesco), which have an international reach and an interest in long-term improvements that extend beyond the usual political cycle (4–5 years) of governments. Some of these companies have reduced their greenhouse gas emissions significantly through using the CFT [55]. If similar emission reductions were replicated across the food chains of these global companies, a significant real-world impact on reducing global food sector emissions could be achieved. Where this can be combined with pro-poor redistributive measures, a significant contribution could be made to the SDGs, especially SDGs 1, 3, 12 and 13.

## 7. Discussion

Finding land management options that co-deliver across a number of global challenges is difficult, but as described in this article, experimental networks [56,57], modelling tools [9,57,58] and spatial analysis [44,45,48,49] are helping to identify potential co-benefits and trade-offs. Frameworks allowing comparisons across ecosystem services [47,59] and across the SDGs [6] also help to identify options that co-deliver to more than one challenge.

Because so many land management options show potential trade-offs, policy-makers need strong evidence to support decisions that they make, and to assess and mitigate the risks associated with those decisions. The science community needs to be ready to provide that evidence. There will undoubtedly be some significant trade-offs in the future between delivering food security, climate change mitigation, biodiversity conservation, the delivery of ecosystem services and of the UN SDGs, but a few options appear to have few negative consequences and could be pursued as ‘no regrets’ with little risk of significant trade-offs. Four such options are discussed below.

*Soil organic matter enhancement* has been proposed to help tackle climate change [15,39,60], as a means of conferring greater resilience to climate change (adaptation), for underpinning enhanced agricultural production [60], and a range of other ecosystem services [59] and SDGs [6,61]. Soil organic matter enhancement is a best management practice that confers multiple ecosystem benefits and is the headline indicator of a number of measures of ecosystem health (such as soil quality and soil health) [6,59], and it can be practised on land without changing land use (i.e. no competition for land) [15]. Increasing soil organic matter content might present a small risk of higher emissions of nitrous oxide in the future (more organic matter means more nitrogen which is a substrate for denitrification when mineralized), but there are few other risks [3]. Increasing soil organic matter confers benefits across a range of ecosystem services [18,59]. Soil organic matter enhancement is promoted under the international ‘4per1000’ initiative, which is a voluntary initiative coordinated by the French Ministry of Agriculture. It focuses on SOC as means to mitigate climate change, while simultaneously improving soil productivity and thus food security. It arose as part of the Lima Paris Action Agenda and is supported by the UN Food and Agriculture Organization (www.4p1000.org). It aims to promote soil organic matter sequestration globally to reach an aspirational target of 4 parts per 1000 (0.4%) annually of the current standing stocks of soil organic matter, through economically viable and environmentally sound agronomic practices [60,61]. The 4per1000 initiative is a policy vehicle through which soil organic matter enhancement can be pursued.

*Ecosystem restoration* has been proposed as a ‘natural climate solution’ [40] delivering carbon sequestration for climate change mitigation, while conferring adaptation co-benefits [62,63]. It also helps to address land degradation [40] and will help to protect or restore biodiversity [64], and promote a range of ecosystem services [43]. The only potential conflict occurs with food security when the ecosystem to be restored is currently being used for food production, for example cultivated tropical and boreal peatlands [65], or restoration of mangroves which may complete with local fisheries and aquaculture [66]. In these areas, trade-offs with food security and rural livelihoods need to be considered, but in most other areas risks are minimal and co-benefits are large.

*Sustainable intensification* involves increasing the productivity of agriculture while minimizing any negative economic, social or environmental externalities [10,23,24]. The critical component of ‘sustainable intensification’ is the ‘sustainable’ part [11]. Intensification of agriculture has delivered greater production, but at the expense of environmental quality [10]. Sustainable intensification therefore needs to deliver increased productivity without the environmental consequences that followed the Green Revolution. There is significant potential for sustainable intensification, with yield gaps for many crops [67] and livestock production systems [33] around the world. Closing these yield gaps will increase food availability, remove pressure from land (potentially leading to land sparing) and will improve sustainable rural livelihoods. While it is unlikely, by itself, to deliver food security or necessary greenhouse gas emission reductions [30], it will contribute positively to all of the global challenges discussed here, support a range of ecosystem services delivered by land and help in the delivery of a number of the UN SDGs. If implemented properly (i.e. truly sustainably), there are few risks associated with pursuing this as a policy goal.

*Demand management* (particularly of unsustainable consumption of food and fibre) is a policy option that, while potentially politically difficult to implement, would provide a range of co-benefits across the global challenges and the SDGs. Livestock production is an extremely inefficient way of delivering food to humans because the calories provided by plants have to first pass through an approximately 10% efficient heterotroph [68]. Furthermore, more than 30% of global crop production is used to feed livestock, rather than people directly [69]. It is not surprising, therefore, that the greenhouse gas footprint of livestock products is approximately 100 times greater than of plant-based foods [70]. Reducing overconsumption of livestock products would greatly reduce the environmental impact of food production [30,31]. Studies show that it is not necessary for humans to become vegetarian or vegan to have significant impacts on climate change and food security—a shift globally towards healthy diets would greatly reduce the adverse environmental impacts of food production [2,30,31,71–73]. Co-benefits between climate, other aspects of environmental impact and human health (particularly through reduction in risk of non-communicable diseases) have also been demonstrated [31,74,75]. Recently, Muller *et al*. [35] examined how far organic farming could go towards feeding the world. The study showed that organic farming, or other lower-impact forms of farming, could make a significant contribution to world food supply, but only if demand for livestock products was dramatically reduced [35]. The main finding from this and other studies examining dietary change and waste reduction is that tackling demand, particularly the current and projected overconsumption of livestock products, greatly reduces pressure on land and creates the ‘headspace’ for other versions of global agriculture and food production to be accommodated. Demand management, through improving human diets and reducing waste, is therefore a policy target that would provide multiple benefits [30,70].

There is no doubt that managing the global land resource to meet the multiple demands expected from it will be extremely difficult, but there are a few ‘no regrets’ options that could be implemented that will provide multiple co-benefits with relatively few risks of trade-offs. While we improve our understanding of the complex interactions between land, food security, climate, environment and sustainable development, enough is known for us to begin to shift policy and to develop tools that allow our best scientific understanding to be used by the land managers and policy-makers who will need to make the change towards a more sustainable future.

## Data accessibility

No original data are presented in this perspective paper.
